# Correlation of positron emission tomography ventilation-perfusion matching with CT densitometry in severe emphysema

**DOI:** 10.1186/s13550-020-00672-8

**Published:** 2020-07-28

**Authors:** Asha Bonney, Carrie-Anne Wagner, Shankar Siva, Jason Callahan, Pierre-Yves Le Roux, Diane M. Pascoe, Louis Irving, Michael S. Hofman, Daniel P. Steinfort

**Affiliations:** 1 Department of Respiratory Medicine, Royal Melbourne Hospital, Parkville, Victoria Australia; 2 Department of Medicine, University of Melbourne, Parkville, Victoria Australia; 3 Department of Radiation Oncology, Sir Peter MacCallum Cancer Centre, Melbourne, Victoria Australia; 4 Department of Molecular Imaging and Therapeutic Nuclear Medicine, Sir Peter MacCallum Cancer Centre, Melbourne, Victoria Australia; 5 Nuclear Medicine Department, University Hospital and EA3878 (GETBO) IFR 148, Brest, France; 6 Department of Radiology, Royal Melbourne Hospital, Parkville, Victoria Australia

**Keywords:** Ventilation, Perfusion, PET, Emphysema, Bronchoscopy

## Abstract

**Background:**

Emphysema severity is frequently measured on CT via densitometry. Correlation with scintigraphic and spirometric functional measures of ventilation or perfusion varies widely, and no prior study has evaluated correlation between densitometry and lobar ventilation/perfusion in patients with severe emphysema. The aim of this study was to evaluate the utility and findings of gallium-68 (^68^Ga) ventilation/perfusion positron emission tomography-CT (^68^Ga-VQ/PET-CT) in severe emphysema assessment.

**Methods:**

Fourteen consecutive patients undergoing evaluation for bronchoscopic lung volume reduction between March 2015 and March 2018 underwent ^68^Ga-VQ/PET-CT assessment for lobar functional lung mapping, in addition to CT densitometry. Correlations between CT densitometry and ^68^Ga-VQ/PET-CT parameters for individual lobar lung function were sought.

**Results:**

CT densitometry assessment of emphysema correlated only weakly (*R*^2^ = 0.13) with lobar perfusion and was not correlated with ventilation (*R*^2^ = 0.04). Densitometry was moderately (*R*^2^ = 0.67) correlated with V/Q units in upper lobes, though poorly reflected physiological function in lower lobes (*R*^2^ = 0.19). Emphysema severity, as measured by CT densitometry, was moderately correlated with proportion of normal V/Q units and matched V/Q defects in individual lobes.

**Conclusions:**

Assessment of lobar pulmonary function by ^68^Ga-VQ/PET-CT provides physiologic information not evident on CT densitometry such as ventilation and perfusion specifics and matched defects. Further research is needed to see if the discordant findings on ^68^Ga-VQ/PET-CT provide prognostic information or can be used to modify patient management and improve outcomes.

## Background

The airflow limitation that characterizes chronic obstructive pulmonary disease (COPD) is caused by a mixture of small airways disease (e.g. obstructive bronchiolitis) and parenchymal destruction (emphysema), the relative contributions of which vary from person to person [1]. Changes of emphysema may be observed on chest computed tomography (CT) and may predate development of spirometric abnormalities [2]. Distribution and severity of emphysema may be visually assessed but is more accurately quantified using CT densitometry which measures the percentage of voxels within the lung that have density below a predefined threshold, usually either − 910 or − 950 Hounsfield Units (HU) [2].

Densitometry has been shown to exhibit moderate correlation with physiologic measures of COPD severity [2], though heterogeneity within and between studies is high, and further research is required. Emphysema quantification by MRI demonstrates lower agreement between anatomic imaging and perfusion abnormalities in severe COPD, compared to mild COPD [3]. Studies using 2-dimensional scintigraphy in patients with severe emphysema indicate that regional ventilation correlates poorly with CT densitometry [4]. Perfusion appears more closely matched with radiologic destruction [5]. Measures of air trapping within individual lobes have also been shown to correlate poorly with CT densitometry [6].

The use of gallium-68 (^68^Ga) ventilation/perfusion (VQ) positron emission tomography (PET)-CT imaging for assessment of regional/lobar lung function, including visualization of changes following endobronchial valve insertion, has previously been reported [7, 8]. This technique allows regional assessment of VQ at a sublobar anatomical level with superior resolution to planar VQ and single photon emission tomography (SPECT) VQ imaging [9, 10]. The study can also be performed with respiratory gating enabling more accurate quantification [11]. Global ^68^Ga-VQ/PET-CT assessments of VQ function are known to correlate with standard pulmonary function tests (PFTs) [12].

In this study, we evaluate the utility and findings of ^68^Ga-VQ/PET-CT in severe emphysema assessment and its correlations with CT densitometry.

## Methods

Consecutive patients undergoing evaluation for bronchoscopic lung volume reduction between March 2015 and March 2018 were considered for inclusion in this study. Patients with COPD with exercise limitation were evaluated according to consensus guidelines [12, 13], with assessment including total lung volume (TLV) and residual volume (RV), quantitative CT chest for determination of fissure integrity [14], and ventilation-perfusion scanning. Correlations between unmatched defects and voxel density, matched defects and voxel density, normal V/Q and voxel density, ventilation and perfusion, perfusion and voxel density, ventilation and voxel density, perfusion and normal V/Q, and ventilation and normal V/Q were assessed for all lobes combined and individually. This was a prospective study approved by the Melbourne Health Human Research & Ethics Committee (QA2017103). Informed consent was obtained from all individual participants included in the study.

### CT acquisition

CT chest imaging, in both inspiratory and expiratory phases, was performed with Siemens Somatom Definition Flash camera (Siemens Healthcare Pty Ltd. Bayswater, Australia) using the following parameters: CARE Dose4D, ref vKp 120, Qref mAs 100, x-ray beam collimation 128 × 0.6, rotation time 0.5 s, and pitch 0.8.

### CT densitometry

Quantitative CT analysis was performed on all scans using the StratX® software (PulmonX, Australia). In each scan, the lungs, pulmonary fissures, and pulmonary lobes were automatically segmented, visually checked, and edited by trained medical analysts [15–18]. The volumes of the lungs and lobes were extracted from the segmented volumes. Emphysema was quantified by attenuation thresholding as the percentage of voxels below – 910 HU. Results were recorded on a lobar basis, with destruction measured according to the percentage of parenchyma within the lobe less than a tissue density threshold of – 910 HU [19].

### ^68^Ga-VQ/PET-CT

Ventilation-perfusion scanning was performed using ^68^Ga-VQ/PET-CT as previously described [11]. The patient was placed in a supine position, and Galligas, prepared by placing ^68^Ga in a Technegas generator (Cyclopharm, Australia), was inhaled. The patient was then positioned supine, with their arms raised on a GE Discovery 690 camera, and a low-dose CT scan was acquired covering the lungs using the following parameters: CARE Dose4D, ref vKp 120, Qref mAs 55, x-ray beam collimation 24 × 3.0, rotation time 0.5 s, and pitch 1.0. A 2–3 bed ventilation PET scan was acquired at 5 min per bed position. While the patient was in the same position, the patient was then administered with approximately 50 MBq of ^68^Ga-macroaggregated albumin (MAA) intravenously, and a perfusion PET scan was acquired at 5 min per bed position.

An experienced nuclear medicine physician completed manual delineation of functional lung volumes using the MIM Encore software (MIM version 6.7) and using methodology we have previously defined [12, 20]. Areas of normal ventilation and perfusion were defined by the nuclear medicine physician by including any lung parenchyma with Galligas for the ventilation contour or Ga-MAA for the perfusion contour. Percentage lobar ventilation, perfusion, and CT volume, as a percentage of total lung values, were recorded, along with normal ventilation/perfusion, matched, and unmatched defects for each lobe. The sum of matched/unmatched defects and normal ventilation was equal to 100%, as previously described [8].

### Pulmonary function testing

Measurement of spirometry, gas diffusion capacity, and total lung volumes were conducted in accordance with the American Thoracic Society–European Respiratory Society guidelines [21].

### Data analysis

Statistical analysis was performed using Microsoft Excel in version 2010 (Washington, USA). Clinical and demographic data are presented using summary statistics. Correlations were sought by Pearson’s correlation coefficient test for non-normally distributed data (*R*_s_) by convention, and *R* between 0.0and 0.2 was regarded as negligible, 0.2–0.4 as weak, 0.4–0.7 as moderate, 0.7–0.9 as strong, and 0.9–1.0 as very strong correlation [22].

## Results

Fourteen patients were included in this study. Mean age was 72 years, with spirometry demonstrating severe airflow obstruction in all patients (Table 1), with mean FEV_1_ 34% predicted (range 19–47%). All demonstrated significant exercise limitation, with median modified Medical Research Council dyspnea score 3 (range 2–3) and 6-min walk distance (6MWD) 280 m (130–440 m).

**Table 1:** Patient demographics

Patient characteristics (***n*** = 14)	Mean	Median	Range
**Age** (years)	72	70	56 – 83
**Male: female**	11:3		
**FEV**_**1**_ Litres % predicted	0.94 34	0.9 35.5	0.5–1.42 19–47
**FEV**_**1**_**/FVC (%)**	31	31	19–44
**DLCO** (% predicted)	40	37	28–64
**Residual volume** Litres % predicted	5.0 197	4.8 184	3.8–7.6 164–271
6MWD (m)	284	280	130–440
mMRC dyspnea score	3	3	2–3
Destruction scores* Upper lobes (%) Lower lobes (%) Most severely affected lobe (%) LUL 36% (5) RUL 21% (3) RML 7% (1) LLL 14% (2) RLL 14% (2) RUL and RML 7% (1)	56 52 69	60 52 73	4–82 19–76 50–82

*Percentage of lung with density below – 910 HU

*FEV*_*1*_ forced expiratory volume (first second)*, FVC* forced vital capacity, *DLCO* diffusing capacity of lung for carbon monoxide, *6MWD* 6-min walk distance, *mMRC dyspnea score* modified Medical Research Council dyspnea score (stratifies severity of dyspnea and associated disability)*, LUL* left upper lobe, *RUL* right upper lobe, *RML* right middle lobe, *LLL* left lower lobe, *RLL* right lower lobe

Densitometry measures indicated high destruction scores with a median voxel density ≤ − 910 HU of 58% (mean 54%) across all lobes. Densitometry demonstrated an overall linear relationship with CT volume in all lobes with a slope coefficient of 0.15, although percentage variance (*R*^2^) was poor at 0.09. This connection was the strongest in the upper lobes with a slope coefficient 0.18 (*R*^2^ = 0.31, *p* value < 0.05).

### ^68^Ga-VQ/PET-CT analysis

**Fig. 1:**
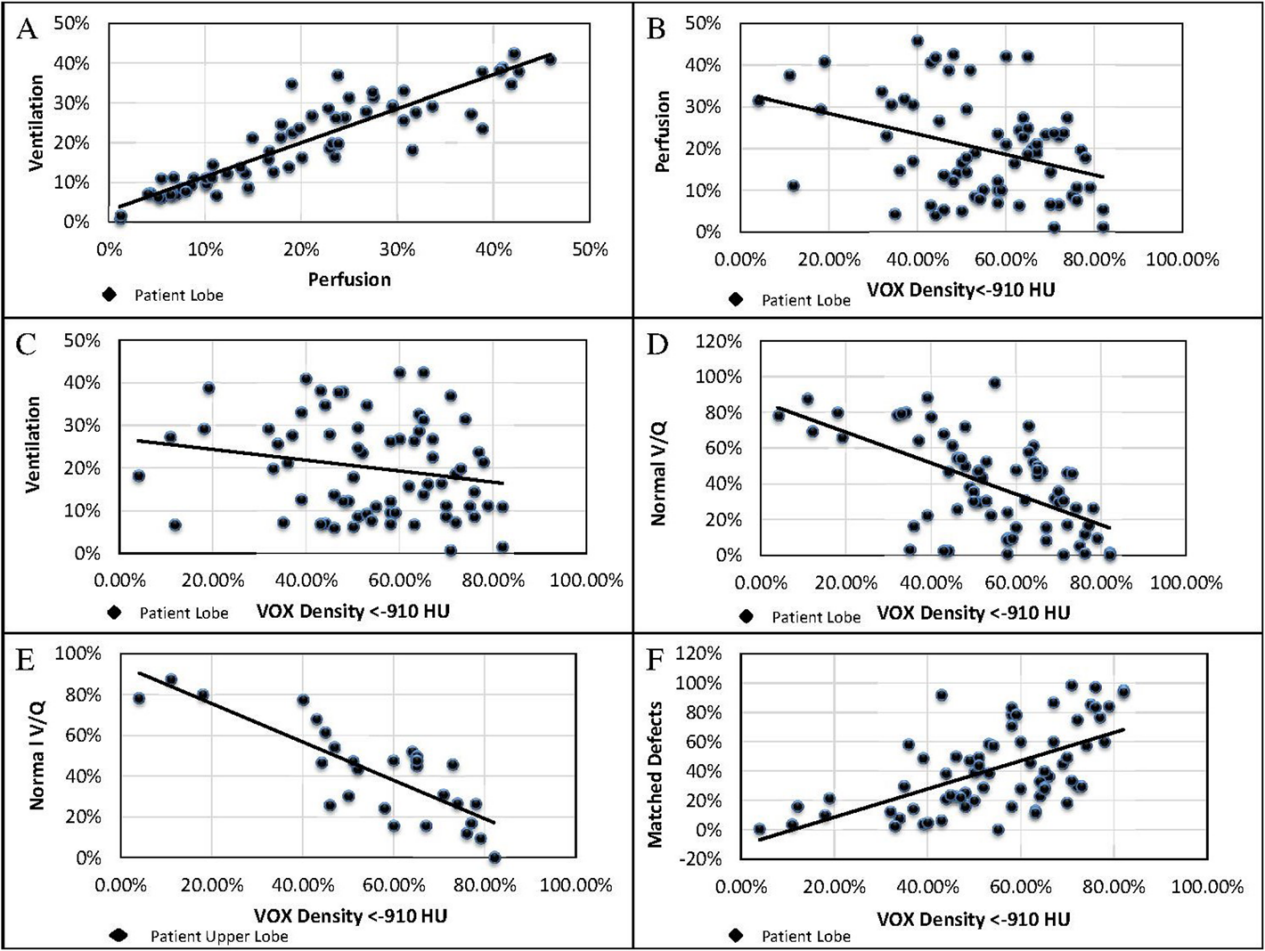
Shown are the relationships between perfusion & ventilation, as demonstrated on 68Ga-VQ/PET-CT and emphysema severity, as demonstrated on CT densitometry. **a**) perfusion versus ventilation in all lobes. **b**) voxel density versus perfusion in all lobes. **c**) voxel density versus ventilation in all lobes. **d**) voxel density versus proportion of lung with normal V/Q units. **e**) voxel density versus proportion of upper lobes with normal V/Q units. **f**) voxel density versus, proportion of lung with matched V/Q defects

Results from comparison of densitometric evaluation of emphysema and lobar function as measured by ^68^Ga-VQ/PET-CT are presented in Table 2. Functional lung mapping with ^68^Ga-VQ/PET-CT demonstrated high internal correlation between perfusion and ventilation (*R*^2^ = 0.82, *p* < 0.0001) (Fig. 1a). High destruction scores on densitometry demonstrated a negligible negative association with perfusion (*R*^2^ = .13, *p* = 0.002) (Fig. 1b), and no correlation with ventilation (*R*^2^ = 0.04, *p* = 0.10) (Fig. 1c). Correlations were stronger for upper lobes than lower lobes (Table 2).

**Table 2:** Voxel density and functional lung mapping

Measure	Site	Slope coefficient	***R***^**2**^	***P*** value
**Voxel density vs normal functional lung**	All lobes	− 0.86	0.33	< 0.05
	Upper lobes	− 0.94	0.67	< 0.05
	Lower lobes	− 0.78	0.19	< 0.05
**Voxel density vs Matched defects**	All lobes	0.96	0.36	< 0.05
	Upper lobes	0.98	0.58	< 0.05
	Lower lobes	1.05	0.34	< 0.05
**Voxel density vs unmatched defects**	All lobes	− 0.10	0.01	0.33
	Upper lobes	− 0.04	0.01	0.68
	Lower lobes	− 0.27	0.07	0.18
**Voxel density vs perfusion**	All lobes	− 0.25	0.13	< 0.05
	Upper lobes	− 0.28	0.26	< 0.05
	Lower lobes	− 0.28	0.17	< 0.05
**Voxel density vs ventilation**	All lobes	− 0.13	0.04	0.10
	Upper lobes	-0.09	0.03	0.35
	Lower lobes	-0.26	0.16	< 0.05
**Perfusion vs ventilation**	All lobes	0.86	0.82	< 0.05
	Upper lobes	0.70	0.67	< 0.05
	Lower lobes	0.86	0.79	< 0.05
**Perfusion vs normal VQ**	All lobes	1.31	0.35	< 0.05
	Upper lobes	1.52	0.52	< 0.05
	Lower lobes	2.15	0.66	< 0.05
**Ventilation vs normal VQ**	All lobes	1.94	0.51	< 0.05
	Upper lobes	1.13	0.20	< 0.05
	Lower lobes	1.94	0.51	< 0.05

Interestingly, densitometry was more strongly correlated with lobar function, as evaluated by ^68^Ga-VQ/PET-CT. Densitometry and percentage normal V/Q units within lobes demonstrated weak correlation (*R*^2^ = 0.33, *p* < 0.0001), with greater correlation seen in upper lobes (*R*^2^ = 0.67, *p* < 0.0001) (Fig. 1d, e). Emphysema severity as measured by CT densitometry also demonstrates weak correlation with the proportion of matched V/Q defects within individual lobes (*R*^2^ = 0.36, *p* < 0.0001) (Fig. 1f). The relationship is again stronger in upper than lower lobes (Table 2).

No correlation was seen between CT densitometry and unmatched defects within the lung (Table 2).

## Discussion

**Fig. 2:**
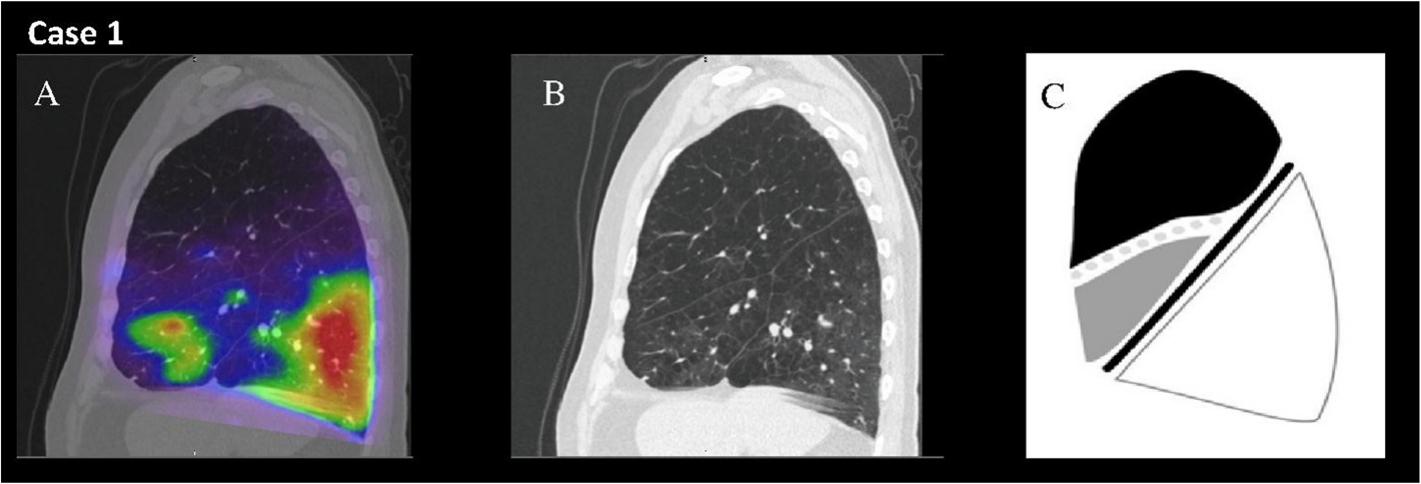
Highlighted are sagittal fused 68Ga-VQ/PET-CT (panel **a**), CT densitometry (**b**), and StratX® report (**c**) images from a participant (case 1) demonstrating an example of concordance of modalities in the upper lobe. Panel A demonstrates perfusion scanning with significantly reduced perfusion to RUL and RML (RUL 11%, RML 6%, RLL 43%). Panel B demonstrates CT appearance, with C illustrating destruction severity, with 79% of RUL, 63% of RML, and 48% of RLL voxels with measured density < − 910 HU)

**Fig. 3:**
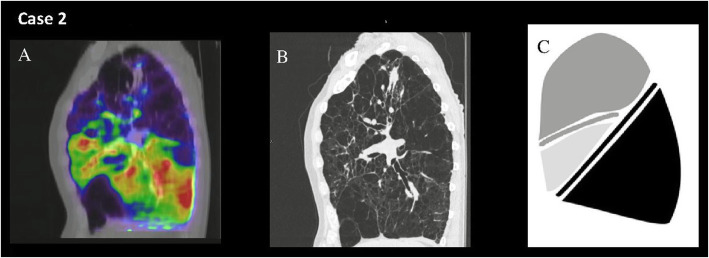
Highlighted are sagittal fused 68Ga-VQ/PET-CT (**a**), CT densitometry (**b**), and StratX® report (**c**) images from a participant (case 2) demonstrating discordance in modalities in the lower lobes, with high perfusion to lower lobe despite high destruction scores based on CT densitometry. Panel A depicts the right lung with perfusion in color (RUL 42%, RML 12%, RLL 14%), with b showing the CT density. Panel C It indicates right lower lobe having the highest destruction score (70%) despite functional imaging suggesting it had an adequate level of perfusion with one of the higher lobar percentages of normal ventilation/perfusion (30%)

Our study demonstrates that lobar functional lung mapping in patients with severe COPD provides physiologic information not evident on CT densitometric analysis (Figs. 2 and 3). Significant inter-individual variability was observed in the relationship between lobar destruction scores and physiologic function, as measured by ^68^Ga-VQ/PET-CT. Relationships between densitometry-assessed severity of emphysema and functional lung measurements were much stronger in upper lobes (compared to lower lobes) for all parameters examined.

CT densitometry is the commonest measure for assessment of emphysema severity. CT density shows a variable relationship to clinically relevant parameters, with studies demonstrating weak-to-moderate correlation with FEV1, DLCO, and symptom score [2]. However, in addition to high heterogeneity within and between studies regarding densitometry and PFTs’ relationship, evidence of publication bias further clouds the exact nature between these findings [2].

Changes in pulmonary blood flow can be observed even in the absence of parenchymal abnormality in COPD [23]. MRI studies have suggested greater correlation with parenchymal destruction (as measured by densitometry) and lobar perfusion [24], though subjects in this study had less severe COPD which perhaps explains the improved correlation between densitometry and lobar perfusion observed in our study. MRI studies also have previously reported only moderate correlation between ventilation and destruction scores [25].

V-Q matching has previously been undertaken with SPECT/CT to allow calculation of lobar function [26]. In this study, we have used a novel technique using PET tracers. PET has significant technical advantages to SPECT including higher sensitivity for detecting radioactive decay, higher spatial and temporal resolution, and superior quantitative capability [27]. For lung imaging, V/Q PET/CT is now possible by substituting ^99m^Tc with ^68^Ga, using the same carrier molecules as conventional V/Q imaging. In a prospective 30 patients study, we demonstrated that the percentage of lung volume with normal ventilation and perfusion > 90% correctly identified patients with COPD in 93% of patients [12]. The high correlation between global measures of lung V/Q and RFTs supports the concept of using ^68^Ga V/Q PET/CT to predict consequences of therapies that affect regional function.

Our findings indicate that ^68^Ga-VQ/PET-CT provides potentially significant information regarding lobar lung function, beyond that identified in routine densitometry assessment of emphysema severity. Functional information in emphysema is likely to be clinically valuable in a number of scenarios, such as for assessment of endobronchial valves, surgical resection, and radiotherapy. While weak correlation was observed between destruction scores and perfusion, as well as normal functional lung units, no correlation could be identified for ventilation, or for proportion of unmatched V-Q defects. Unmatched defects identified by VQ-PET were demonstrated in prior studies to be equally comprised of mis-matched defects (V > Q) and reverse mis-matched defects (V < Q) [12]. Thus, assessment of lobar function through CT and perfusion studies alone is unlikely to adequately determine lobar function.

## Limitations

This study is limited in size and examines only patients with severe emphysema. Relationship of functional indices with densitometry findings is likely to differ in mild COPD and in those with normal pulmonary function. Whilst association between densitometry and functional lung assessment is stronger in the upper lobes, the exact reason is unclear. We postulate that this may in part be due to ^68^Ga-VQ/PET-CT being a slower acquisition scan, resulting in respiratory motion artefact in the bases. Previous studies have demonstrated upper lobe predominant emphysema which has a stronger negative correlation with pulmonary function testing and a steeper rate of decline over time compared with lower lobe predominant emphysema [28–30]. Although upper lobes are generally the more clinically targeted regions for lung volume reduction, this finding may signify the importance of V/Q assessment in prior to intervention particularly in the lower lobes.

## Conclusion

^68^Ga-VQ/PET-CT provides additional functional information in patients with severe emphysema which may augment CT-densitometric assessment of emphysema severity. Correlation between CT-based destruction scores and functional measures of individual lobar function vary from negligible to moderate. Relationships between destruction scores and physiologic function are uniformly stronger in upper lobes compared to lower lobes. Decisions regarding therapeutic interventions in targeted lobes in severe emphysema patients could be strengthened with the use of both CT and VQ studies available, particularly in unclear cases such as significant functional limitation despite only mild radiological emphysema and decision between multiple potential target lobes for endobronchial valve insertion.

## Data Availability

The datasets used and/or analysed during the current study are available from the corresponding author on reasonable request.

## Abbreviations

^68^Ga: Gallium-68
^68^Ga-VQ/PET-CT: Gallium-68 ventilation/perfusion positron emission tomography computed tomography
6MWD: 6-min walk distance
COPD: Chronic obstructive pulmonary disease
CT: Computed tomography
DLCO: Diffusing capacity of lung for carbon monoxide
FEV_1_: Forced expiratory volume (first second)
FVC: Forced vital capacity
HU: Hounsfield units
mMRC: Modified Medical Research Council
PET: Positron emission tomography
PFTs: Pulmonary function tests
*R*^2^: Percentage variance
RV: Residual volume
SPECT: Single photon emission tomography
TLV: Total lung volume
VQ: Ventilation/perfusion
